# Are we prepared for the future? A mixed-method study on quality management in decentralized family medicine teaching

**DOI:** 10.1080/10872981.2021.1923114

**Published:** 2021-05-11

**Authors:** Roland Koch, Marie-Theres Steffen, Julia Braun, Stefanie Joos

**Affiliations:** Institute for General Practice and Interprofessional Health Care, Tübingen University Hospital, Tübingen, Germany

**Keywords:** Medical education, quality management, family medicine, Germany, decentral learning environments, mixed-methods, qualitative research, quantitative research, survey, interviews

## Abstract

In Germany, two-week clinical clerkships in university-associated general practices have existed since 2002. Approximately 10,000 medical students participate in these decentral clerkships each year. Empirical information on quality management strategies in decentral learning environments is sparse. This nationwide study aims to describe the current quality management efforts of German family medicine departments in response to negative events. A nationwide three-part mixed methods study on the quality management of family medicine clerkships was conducted. First, individuals from n = 37 family medicine departments involved in the organization of family medicine clerkships were interviewed. Interview transcripts were analyzed with qualitative content analysis. Second, a questionnaire on quality management of decentralized learning environments based on the categories of the analysis was developed and sent to the departments. Three negative event cases in family medicine clerkships were included in the questionnaire. Third, interview and survey data were integrated based on respondents’ process descriptions of how each department handled the cases. Of the 37 contacted departments, n = 12 (32%) performed an interview. Major categories of negative events included problems in the student-teacher interaction, didactical challenges, and problematic student behavior. Twenty departments answered at least one questionnaire (54%). Most respondents indicated that their department conducts quality management in decentralized teaching. Negative events in decentral family medicine clerkships occurred at a rate of 66.4 to 179.5 events per 10.000 Students per semester. The mixed-method analysis showed that departments are conscious about quality management issues in decentral learning environments but adhere to heterogeneous local standards. Negative events occur regularly in decentral learning environments. Local quality management processes exist but lack national harmonization. Further outcome-based research is needed to explore the effectiveness and feasibility of quality management strategies. This will become increasingly relevant with an expected upscaled family medicine content.

## Introduction

Even after 30 years, Shipengrover and James’ term ‘Black box’ still fittingly describes the difficulties of quality management (QM) in decentral learning environments [1,2]: Decentral learning environments such as GP practices are independent entities with unique values and team dynamics, clinical mission and scope, administrative and/or legal procedures, communication pathways and different teaching methods [2]. Decentral course managers are tasked with managing teaching quality without having much insight into or access to the ‘Black Box’.

At the same time, decentralized training provides unique opportunities for learners [3]: In decentral learning environments [4], medical students can learn about different levels of health care and intersectoral cooperation [5,6]. Following Kolb’s learning theory, learning is a dynamic transaction between learners and the learning environment [7]. In GP practices, the learning environment includes the setting (facilities, location), patients, the GP teacher, and the health care team. GP teacher and medical student engage in a 1:1 teaching relationship that can be rewarding [5,6,8] or exposes the learner to possible mistreatment or other negative events [9].

QM efforts in medical education approach such negative events in two ways: One, by implementing structures to prevent their occurrence. Second, by establishing processes to adequately handle such events and to possibly learn from them. Fleit [10] and Smith-Coggins [11] recently explored QM processes on negative event reporting. The authors demonstrated that the implementation of faculty-wide incident reporting systems positively affected student experiences and initiated an organizational transformation. In Germany, there is no widely acclaimed national standard for reporting or preventing negative events in decentral learning environments such as the obligatory family medicine clerkship, even though around 10,000 medical students attend the clerkship each semester [6,8].

In 2025, a reform of the medical licensure act [12] is expected for Germany. The family medicine clerkship period will be extended from two to eight weeks. Furthermore, students will be allocated to the same teaching practice during their studies. Concerning negative events and their reporting, this has several implications: a) the possibility of events will increase, b) possible incidents can and should be followed up longitudinally and c) QM measures must consider effective prevention of negative events in GP practices. This study aims to describe current efforts in reporting and handling negative events that occur in decentral learning environments by family medicine departments in Germany and to derive ideas for their prevention.

## Methods

This study was carried out as a nationwide mixed-method study in three parts:

### Study part one: qualitative expert interviews

The first part of this study was conducted as part of a larger qualitative interview study with teaching physicians, students, and experts (Teaching coordinators, heads of department, non-medical teaching staff) in 2017/18. While the student and GP-teacher group were limited to the University of Tübingen, the expert interviews were conducted nationwide. For the expert interviews, all 37 family medicine departments in Germany were contacted by email and asked to participate in semi-structured telephone interviews. The interviews were conducted by JB after consent to participate was obtained. An interview guideline was used which is included as a supplement (Supplement 1). Audio recordings of the interviews were transcribed by JB and MTS. Qualitative content analysis [13] was applied to the transcripts, supported by a software tool (f4 analyze, Dres Schmidt gmbh, Marburg, Germany): JB first separated three interviews of each stakeholder group into code units. She then paraphrased and condensed the code units into subcategories that comprised several code units [13]. JB and RK grouped the subcategories inductively into categories [13]. That way, a coding frame was developed. JB and RK coded three new interview transcripts using the coding frame. The coding results were compared. Discrepancies were discussed and the coding frame was refined by writing descriptions and definitions of the categories and renaming them if needed. JB then applied this coding frame to all interview transcripts.

### Study part two: cross-sectional survey

A questionnaire was newly developed for this study by MTS and RK based on the results of the qualitative analysis in part one. It is included in an English language version as a supplement (Supplement 2).

Questionnaire Items and their dimensions were informed by the categories and subcategories of the coding frame. Respondent gender and age were not asked to ensure anonymity. The questionnaire contained questions about the respondent and his or her position within the department, department size, number of GP practices and students supervised, didactical training for GP’s and questions about current organizational challenges. Furthermore, three case examples that represented the three major problem categories in decentral teaching were integrated into the questionnaire. These cases were derived from the interviews. They provided a standardized context for free text answers in which survey respondents were asked to describe local problem management strategies and processes (see Table 1).

**Table 1.** Case examples developed from narratives in the interviews

**Case example 1: Problems in student-teacher interaction** A student calls you after the family medicine clerkship and reports that her GP teacher made derogative, unfair, and unmotivated comments about her in front of the nursing team. There had been sexualized comments. You are unaware of past negative feedback on the teacher. To your best knowledge, the teacher is engaged, constructive, and popular among colleagues and students.
**Case example 2: Didactic problems** On the 3rd day of the family medicine clerkship, a student writes an angry email complaining that he has no opportunity to work independently whatsoever at his current teaching practice. The owner of the practice and teaching doctor has been around for a long time. His teaching performance is rather mediocre. Several student feedbacks indicate that independent work is hardly possible in this practice. The student wishes to change practices.
**Case example 3: Problematic student behavior** A dedicated teacher, who has not been with your department for very long, calls you during the clerkship. She reports that her current student is acting irrationally. He has already used up his allowed absence time. Furthermore, he keeps leaving the room during the patient consultations. He seems absent-minded in his contact with the nursing staff and during patient consultations. He avoids eye contact and has already been late several times. She is seriously concerned about the student’s health. She is unsure if the student can even be certified as having successfully participated.

The questionnaire was piloted by MTS in a sample of 5 teaching coordinators using the ‘think aloud’ method [14]: The teaching coordinators were presented with the questionnaire and completed the items. While doing so, they were asked to express their interpretation of the questions and to elaborate on their answers. This led to minor changes in wording and the reduction of categorized items in favor of more free-text answers due to the heterogeneity of problem-solving strategies in the pilot sample. The relevance of the case examples was confirmed by all participants of the pilot sample.

Two questionnaires were sent to each of the family medicine departments. Departments were asked to provide one copy to administrative staff and one copy to the head of teaching or the medical director. Survey data were analyzed statistically using SPSS Version 26 (IBM, New York, USA). Descriptive statistics were calculated by MTS and RK. Differences between the two respondent groups were calculated using Fisher’s exact test by MTS and RK. Free text answers were categorized thematically, using the category system developed in step 1 as a template.

### Study part three: integrated analysis of qualitative and quantitative data

In the third study part, the local problem management strategies and processes in response to the three presented negative event cases were translated into flow chart diagrams by a study assistant (EF) not previously involved in the interviews or survey. The mixed-methods analysis integrated the flow charts with study data from the first two study parts (RK): Interview excerpts were used to illustrate how course management argued decisions. The reporting frequency of defined process steps in the survey was presented with pie chart diagrams. The resulting visualized processes and interview excerpts were discussed between the authors (RK, MTS, JB, and SJ). Discrepancies were solved by consensus in choosing appropriate interview excerpts and optimizing the visualization of the flow charts.

Inclusion criteria were: Members of German family medicine departments (heads of teaching or administrative staff). There were no exclusion criteria. Factors that might influence the interviewer such as a personal or mentorship relationship to the interview partner were checked before the interviews.

## Results

### Study part one: qualitative expert interviews

In study part one, 12 Interview partners from different departments were recruited, representing 32% of all departments. Six respondents were female. The mean age was 50.5 ± 7.8 years. Four respondents identified as medical directors, 6 as teaching coordinators, and 2 as administrative staff. The average interview length was 30 minutes which resulted in about 8 pages of text per transcript.

Two main categories were identified in the interviews: Student-teacher interaction from the experts’ perspective and organizational aspects of the clerkship. The categories and subcategories were related to three main problems in decentral teaching: 1) Personal problems between teacher and student including student mistreatment; 2) Didactic problems or suboptimal learning environments and 3) Problematic student behavior. The category system and its subcategories are included as a supplement (Supplement 3).

### Study part two: cross-sectional survey

In the second study part, n = 29 survey participants responded. Twenty departments answered at least one questionnaire (54%). Two questionnaires (3%) did not contain data and were excluded from the analysis. Eleven respondents worked in an administrative position and n = 16 in a management position (Medical director, course coordinator, head of teaching). Characteristics of respondents and their departments are presented in Table 2. Except for free text answers, responses were checked for differences between the two respondent groups using Fisher’s exact test. With one exception, no significant differences between the two respondent groups were found.

**Table 2.** Summary of Survey results

Variable	Valid	Item	Count	%valid	Sig^3^
**Respondent characteristics**					
Respondent position at department	27	Management+	11	41%	n/a
		Administrative staff	16	59%	
Years of employment at department	27	0–3 yrs	7	26%	n.s.
		4–6 yrs	11	41%	
		7–10 yrs	6	22%	
		>11 yrs	3	11%	
**Department characteristics**					
Employees	27	1–5	3	11%	n.s.
		6–10	10	37%	
		11–15	3	11%	
		16–20	3	11%	
		More than 20	8	30%	
Age of department (For how many years has your family medicine department been with an independent chair?)	25	Less or equal 10 years	11	44%	n.s.
		More than 10 years	11	44%	
		No chair established (yet)	3	12%	
Number of associated teaching practices	27	1–50 GP practices	0	0%	n.s.
		51–100 practices	9	33%	
		101–200 practices	10	37%	
		201–300 practices	7	26%	
		301 practices and above	1	4%	
Students supervised in block internships per semester	26	1–50 Students	1	4%	n.s.
		51–100 Students	0	0%	
		101–200 Students	20	77%	
		201–300 Students	4	15%	
		301 Students and above	1	4%	
**Quality management (QM) in decentral teaching**					
Actively involved in QM	27	Yes^1^	26	96%	n.s.
QM Standard (Free text)	26	Standard provided by faculty	3	11%	
		ISO standard	2	8%	
		Don’t know	2	8%	
		System accreditation	1	4%	
		No specified standard	18	69%	
What are you most satisfied with within your institution^4^ in terms of the organization of teaching? (Free text, multiple answers possible)	27	Workflow at the institute	8	30%	n/a
		Support by faculty	7	26%	
		Specific learning environment	7	26%	
		Cooperation with teaching physicians	7	26%	
		Other	14	52%	
		No response	4	15%	
What area of your organization of teaching needs improvement? (Free text, multiple answers possible)	27	Decentralized QM	8	30%	n/a
		Staffing resources	5	18%	
		Digitization	4	15%	
		Other	16	60%	
		No response	3	11%	
**Qualification of teaching practices and GP teachers**					
Didactic standard for teaching practices	27	Yes^1^	22	81%	n.s.
Didactic standard publicly accessible	22	Yes^1^	9	41%	**<0.01**
Frequent didactic training for teachers	27	Yes^1^	20	74%	n.s.
Frequency of didactic training for GP teachers	20	1x/year	3	15%	n.s.
		2x/year	11	55%	
		3 or more x/year	6	30%	
Obligatory	20	Yes^1^	11	55%	n.s.
Performance assessment for students	26	Yes^1^	18	90%	n.s.
**Negative events in decentral training**					
Do you systematically collect feedback from students about negative events during the family medicine clerkship?	27	Yes^1^	23	85%	n.s.
Does it happen that students contact you because of negative events in family medicine clerkship?	27	Yes^1^	26	96.3%	n.s.
Student preference when taking contact (Free text)	26	Email	20	77%	n/a
		Personal	18	69%	
		Telephone	13	50%	
		Via evaluation	4	15%	
How many times do students contact you during the semester due to negative events? (Minimum)^2^	24	0	3	13%	n.s.
		1	11	46%	
		2	7	29%	
		3	2	8%	
		4	1	4%	
How many times do students contact you during the semester due to negative events? (Maximum)^2^	24	1	4	17%	n.s.
		2	6	25%	
		3	9	37%	
		4	4	17%	
		5	1	4%	

^1^unless stated otherwise, the remaining valid responses were ‘no’. ^2^ This variable was a free text variable in which most respondents gave a range (e.g. 2–4 times). The minimum and maximum variables represent the first and the last digit in these ranges, respectively. ^3^ Tested for significant differences between Administrative and Management personal; Fisher’s exact test was used. ^4^ The institution was defined as ‘the family medicine department or institute with an affiliated chair at your university’.

n.s., Not significant; n/a, not applied.

The number of teaching practices associated with each department ranged from 50 to 300 and more. Most departments supervised a student body of between 101 and 200 students per semester (n = 20).

Most survey respondents reported active involvement in QM of decentral learning environments (n = 26). QM was defined as ‘structured regular measures which aim to influence measurable results (e.g. student evaluation)’ (See supplement 2). Only six respondents specified a QM standard (DIN ISO or similar n = 3, local faculty standard n = 3). Systematic evaluation and feedback from students were reported by n = 23 respondents. Respondents were most frequently satisfied with their department’s workflow (n = 8) and the cooperation with teaching physicians (n = 7). Decentralized QM was most frequently stated as needing improvement (n = 8).

Nearly all respondents (n = 26) reported being contacted by students at least once per semester due to negative events during the clerkship. Negative events were defined as ‘e.g. non-compliance with teaching standards, errors in content, unfriendly treatment by the teacher’ and thus included any of the categories of the negative event cases (See supplement 2). Preferred means of contact were via email (n = 20), personal meeting (n = 18) or telephone (n = 13).

The occurrence of negative events during the family medicine clerkship was reported by n = 24 respondents. The frequency ranged from a minimum of 1 (mean value of all reported minima) to a maximum of 3 (mean value of all reported maxima) events per semester. Considering the mean number of students supervised by each respondent as a quotient, the frequency of occurrence of such events was calculated at a minimum of 66.4 to a maximum of 179.5 per 10.000 Students per Semester.

Twenty-two respondents stated that a didactic standard for teaching practices was established at their department. In n = 9 of these cases, respondents stated that the standard was publicly available. This is the only point that showed a significant difference between administrative staff and management staff – only the latter responded positively. Frequent (2 times/year) didactic training courses for teachers were held according to n = 20 of the respondents. Courses were reported as obligatory by n = 11 respondents.

### Study part three: integrated analysis of qualitative and quantitative data

Most respondents (n = 26) described their department’s processes in response to the three negative event cases.

### Case 1: personal problem between teacher and student

**Figure 1.**
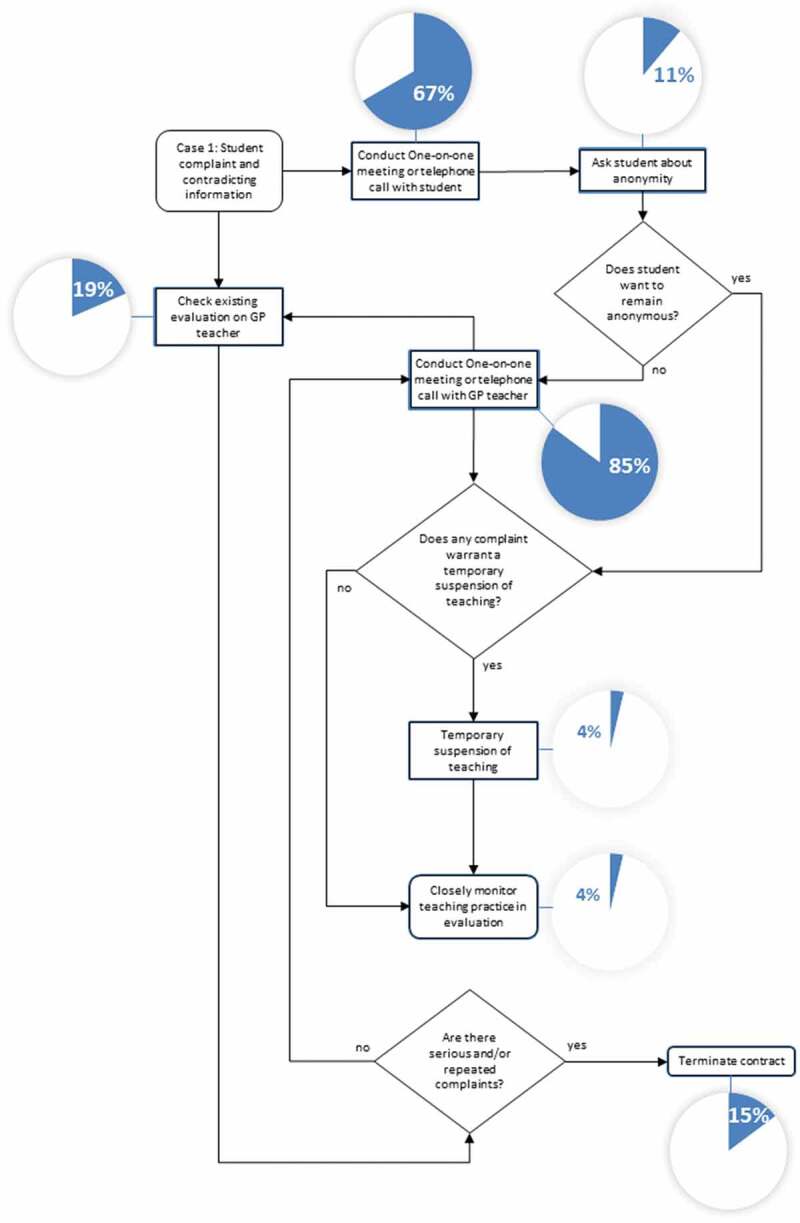
Meta-process and survey reporting frequency of case 1 (Problems in student-teacher interaction)

The first case (see Table 1, case 1) involves both the unfair treatment of the student and contradictory information about the teacher. Figure 1 represents the meta-process of these free-text answers. The pie charts show the proportion of the 26 respondents who named the corresponding process step. Interview excerpts are shown in quotation marks. The interview partner is indicated by the abbreviation EX (Expert), the number of the interview partner (for example EX-1), and the paragraph in the interview transcript (for example 16).

Most respondents stated that both GP teacher and student are interviewed before further steps are considered. If the prior evaluation shows several negative reports and the practice is unwilling to change their behavior towards students, consequences for the GP teacher are considered.

‘[…] if we hear feedback several times that goes in the same direction, we would then contact practices and try to ask why such problems occur and of course we would also try to remedy the problems. If they cannot be turned off, which we have gone through several times in the past, we would part with such a teaching practice.’ (EX-1, 16).

In very severe cases of mistreatment like sexual harassment, contracts are terminated promptly:

‘[referring to an event of sexual harassment] … of course I cannot wait or collect further evidence. So I directly called the teacher. […] he is not a teacher anymore … that is logical, one cannot tolerate that.’ (EX-12, 51)

However, not all such cases warranted contract termination:

‘So we decided not to send female students there anymore … the student wanted to know what the consequences were and we told her. We said: “we do not doubt you, this is what you experienced and our reaction is that none of your female peers will be exposed to that ever again[…]”’ (EX-5, 71–72)

Seldomly and in less severe cases, a partial suspension of teaching is considered.

‘ … when I conducted site visits, I realized that this practice is not suited for teaching. Most of the time, I do not terminate the teaching contract but reduce student allocation, so that they only get one student per year. I do not want to risk running out of teaching practices. Only very severe events will trigger contract termination’ (EX-5, 31)

The last three mentioned consequences of negative events were rarely stated in the process descriptions (less than 10% of responses, see Figure 1).

### Case 2: didactical problem

**Figure 2.**
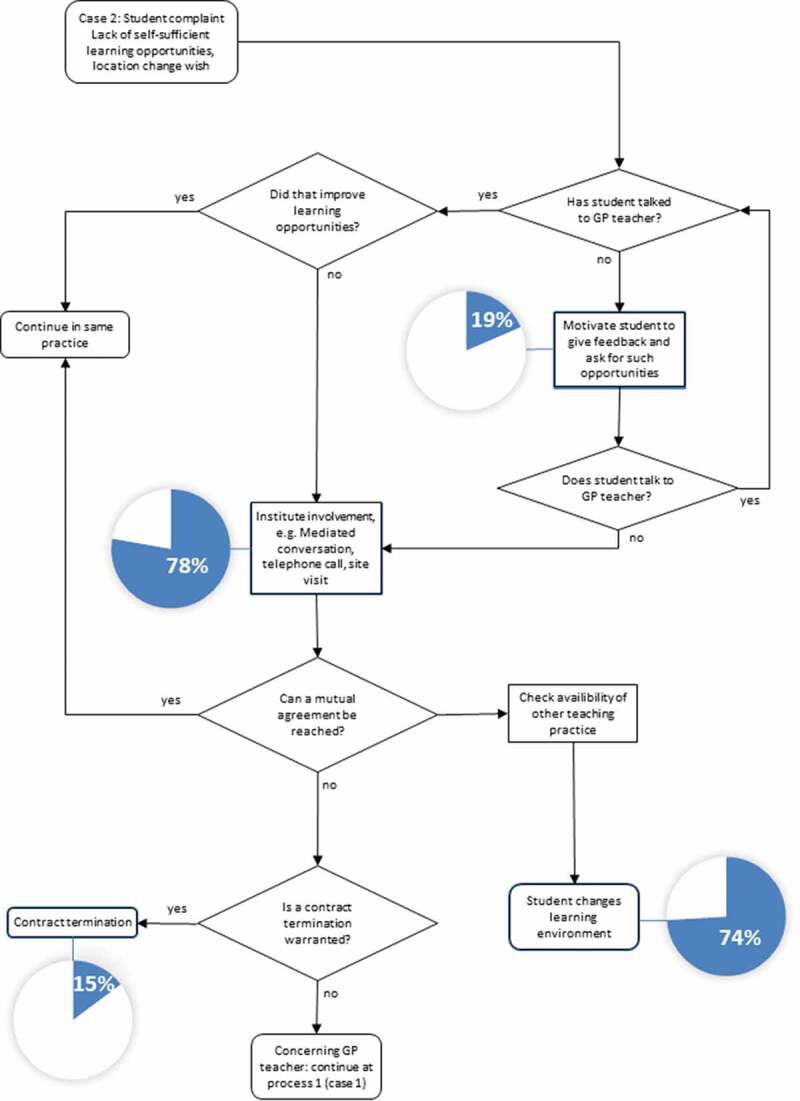
Meta-process and survey reporting frequency of case 2 (Didactic problems)

In the case of didactic problems (see Table 1, case 2), most respondents favored that teacher and student initiate feedback in the decentral learning environment. The process is presented in Figure 2 below.

The next steps depend on whether the decentral feedback between teacher and student had a positive impact on the learning environment. According to the interviewed experts, teachers have different capabilities of taking advice and feedback. If student feedback has no positive impact on the learning environment, the department mediates between teacher and student.

‘It has happened that students repeatedly negatively addressed the same topic. We spoke to the teacher, but he could not see it that way (EX-6, 23).’

If teachers are not receptive to feedback, this is treated as a problem on the teacher’s side.

‘ … in doubt, we would contact the GP practice and […] visit them and ask: “What is going on? Can you change that? Do you realize that students expect to work independently and want to contribute? Do you know they can expect feedback and that it should be given to them?”. If there are repeated reports or the teacher does not understand, then we consider the termination of the contract.’ (EX-2, 20)

### Case 3: problematic student behavior

**Figure 3.**
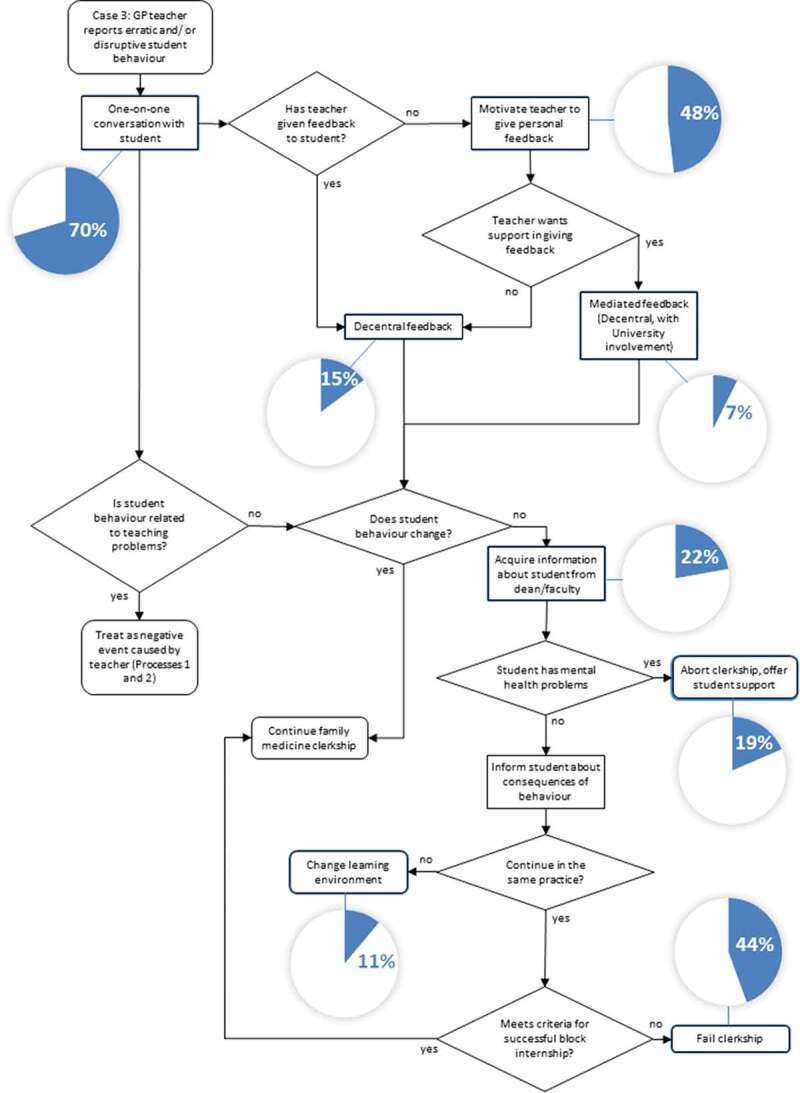
Meta-process and survey reporting frequency of case 3 (Problematic student behaviour)

Examples of problematic student behavior (see Table 1, case 3) included appearances, but also attitudes towards learning and/or patients and staff. The process is presented in Figure 3 below.

‘a teacher reported that he finds it intolerable when a student arrives in a crumpled coat, chews bubble gum, and makes flippant remarks towards patients’ (EX-6,46)

‘There are cases when someone comes, puts his hands in his pockets, and signals with his expression: ‘just leave me alone! I don’t want to work here!’ (EX-7, 49)

The process involves three parts: First, feedback is given by the teacher to the student – either mediated by the department or personally. Next, student feedback is considered (since the student’s behavior might be a consequence of the teacher’s actions). According to the interviews, this is hard to objectify, since teachers’ and students’ perceptions might diverge:

‘90% of the cases, the perception of the teacher does not correspond to the student’s perception. We have experienced several times that the student felt ignored and treated arrogantly […]. And then we had a conversation with the teacher and he said: “Yeah, well, he did not ask anything, he was late, he didn’t have a clean coat, he was unkempt and he stood there listlessly”’ (EX-10, 28).

Third, if the student is identified as a source of the problem, he or she is treated in cooperation with the faculty.

‘Yeah, we’ll call them in and talk to them, and then we’ll sort it out. And it’s always settled. As I said: Most of the time, those who exhibit problematic behavior are usually challenged with particularly difficult personal situations. The one student I told you about, he went into therapy, and one year later, he did a very successful clerkship’. (EX-3, 42)

If mental health issues are ruled out, the student is warned about possible consequences.

Depending on local standards, the student then either passes or fails the clerkship and must repeat it in a different practice later.

## Discussion

Most family medicine departments have local processes available in response to common negative events in GP practices. Most interview partners and survey respondents describe themselves as actively involved in QM. While there is consciousness about negative events in family medicine clerkships, standardized QM processes to handle them are based on local strategies and/or values of the respondent. QM measures to prevent negative events were mostly restricted to GP teacher didactics training. It became evident that QM processes in response to negative events in decentral learning environments hardly are aligned nationally. The following segments discuss the major findings of the integrated analysis.

### QM at different levels: central and decentral

There is a distinction between what is happening in the GP practice (decentral) and at the university (central). In the case of didactical problems (case 2) and student behavior (case 3), the process allocates some responsibility to learners and teachers locally. However, department or faculty involvement (central actions) is more commonly reported than decentral problem-solving. The involvement of departments is even more common if student mistreatment (case 1) occurs. Departments collect data on teaching practices, but there is little transparency on its use in QM.

There are several possible explanations for these observations. For one, universities depend on the goodwill of teaching practices. Family medicine in Germany heavily relies on decentral teaching and teaching contracts are voluntary. Thus, reprimanding or disciplining a teaching practice can lead to an undersupply of teaching practices due to the cancellation of contracts by teachers. The sometimes artificial distinction between centralized and decentralized teaching risks being leavened by questions of teaching quality. Here, decentral teaching risks unreflectively being called suboptimal teaching in contrast to teaching at university hospitals [15]. These aspects contribute to the course management and the central university being positioned as a supervisory authority rather than partners of decentral teaching environments [16].

The data also shows ways to traverse these barriers between central and decentral teaching: Seldomly, the department is involved as a mediator between teachers and students or conducts site visits, thus reaching out locally. Herein lies the chance to develop new QM strategies for decentral learning environments.

### Escalation of problems in decentral teaching

The processes correspond to a problem hierarchy that ranges from didactical challenges and problematic behavior to student mistreatment or psychiatric problems. Each negative event case presented to the respondents (unfair treatment, didactical problems, and erratic student behavior) warrants its process, and the processes can escalate in response to the severity of the problem.

The low reported frequency of contract termination or suspension of teaching mirrors a low reported frequency of severe incidents like student harassment. This may be due to a low prevalence of such incidents in family medicine clerkships or due to underreporting [9].

Nevertheless, in terms of problem escalation, survey respondents and interview partners adhere to either local faculty standards or his or her code of conduct. This leads to different escalation strategies in different locations and very different student experiences if such events occur. A clear problem hierarchy would be a good start for the standardized reporting of events. Smith-Coggins implemented a transparent faculty-wide problem hierarchy with accurate descriptions of negative events. Severe negative events spurred rigorous consequences. The reporting of events, in turn, reduced student mistreatment [11]. Based on that example, a national, standardized online reporting form could be developed and made accessible to both teachers and students, acknowledging that both perspectives are equally important.

### Are we prepared for the future?

Based on our results, there is consciousness about the importance of QM of decentral teaching in German family medicine departments. However, appropriate tools to engage in a nationally harmonized development of QM in this specific setting are missing.

As previous cases of the implementation of negative event reporting have shown, there are several ways to collect data [10,11]. The results from the survey show departments’ preference for personal meetings. While these meetings have clear advantages, they have a high threshold. Students preferred digital means (email) or telephone as a first contact. Thus, standardized digital reporting forms should be implemented following Fleit’s example [10]. They have the advantage of working ad-hoc, providing enough anonymity, and ignoring physical distances. Teachers should also have access to the reporting forms, either for self-reporting or for reporting problematic student behavior.

Collecting and checking information about learning environments must not be a ‘behind the scenes’ action. If not communicated correctly, it causes trust issues, adding to the needlessly high reporting threshold both for teaching practices and students. To counteract this, rules and QM processes must be transparently communicated to all stakeholders [10,11]. Nationally acclaimed standards will help this communication and ease the burden on course managers [16].

The preparation of students and teachers alike seems to be the most powerful tool for the prevention and handling of negative events in decentral learning environments. Even if the ‘black box’ remains, a standardized process comprising the education of learners and teachers can ensure that the input into the ‘black box’ follows quality standards [1]: Students need to be perceptive of mistreatment and didactical shortcomings and trained in giving feedback to their preceptors. Preparing students to give adequate feedback and how to deal with possible problems in decentral 1:1 teaching settings is as important as preparing students for medical tasks [11]. Teachers on the other hand must know the exact definition of mistreatment and be sufficiently trained to accept feedback, give feedback, and reflect on how to improve. If needed, course management can mediate between the parties.

With time, training both recipients and senders of feedback can achieve a cultural change and impact the whole organization. This approach to quality is ‘quality as transformation’ according to Harvey and Green [17]. Through a continuous process of transformation based on feedback, core values, and a common goal, family medicine departments can overcome the obstacle of a ‘black box’ in decentral learning environments.

Several obstacles are not sufficiently understood: The impact of personal experience and power dynamics in decentral learning environments and how organizational culture affects teachers and learners, for example. Future research should address this with an appropriate methodology (e.g. narrative analysis). Furthermore, different approaches to QM in decentral learning environments should be implemented and empirically evaluated.

In summary, tools to improve QM in family medicine teaching, with its large proportion of training in decentralized environments, include low-threshold reporting of negative events, comprehensible and transparent escalation steps when events occur, improvement of faculty and student competencies to prevent negative events, mediation of student-teacher interactions, and faculty and student participation in the development and evaluation of QM measures.

## Strengths and limitations

The nature of the interviews and survey (self-reporting) limits the results of this study due to reporting bias. GP teacher and student interviews were not considered for the results section because those interviews were restricted to the University of Tübingen. Future studies should also include student and family practice perspectives in addition to the centralized departmental perspective. The focus on course managers and the methodological choice of qualitative analysis did not allow an in-depth analysis of hierarchies, power dynamics, or intrapersonal experiences. Nevertheless, the method and data source were suited to describe the national context.

In study part two, the response rate to the questionnaire was mediocre. Even though reminder emails and calls were conducted, our results represent only half of the German family departments. A survey of non-respondents would have provided information on why departments choose not to share information on QM. The inclusion of both administrative and management staff was a deliberate choice to achieve a higher return rate, assuming that the tasks were differently distributed across both groups. No significant differences were found between the groups, which allows for a combined analysis of both groups. Despite these limitations, it can be assumed that departments already more involved in QM issues have responded, i.e. that the problem is rather underestimated in the present study.

Methodically, the integration of qualitative and quantitative data in study part three allowed an amalgamation of representative processes and decisions, problem hierarchies, and levels. This mixed-methods approach allowed to combine an in-depth perspective on decisions and certain components of the processes with the generalizability of those results. However, the development of flow charts based on empirical data is a novel approach. The stated answer frequencies should not be regarded as ‘outcomes’ or prevalence, but as a statement on how known a measurable outcome is in the process of QM. In that regard, the empirical mixed-methods approach provides a ground for a broader discussion of this important topic.

## Conclusion

Our study revealed two major points: Negative events occur in decentral learning environments. These are especially vulnerable scenarios. While there is a consciousness about the problem, the lack of standardized QM processes remains a nationwide challenge.

To meet this challenge in the German medical curriculum with an expected upscaled family medicine content, scientifically sound, prospective implementation studies must be carried out in this specific setting. The status quo described here provides the groundwork for such endeavors.

## Supplementary Material

Supplemental Material

## Data Availability

The datasets generated and/or analyzed during the current study are not publicly available due to protecting respondents and interview partners but are available from the corresponding author on reasonable request.

## Abbreviations

GP, General Practitioner; QM, Quality Management; EX, Expert (interview partner).
