# Supplementary material for: Are we prepared for the future? A mixed-method study on quality management in decentralized family medicine teaching
Source: Med Educ Online. 2021 May 11;26(1):1923114. doi: 10.1080/10872981.2021.1923114 (PMC8118471; doi:10.1080/10872981.2021.1923114)
Supplement: Supplemental Material [file ZMEO_A_1923114_SM9130.zip › Supplementary files/Supplement 1 Interview Guideline Experts.docx]

Single interviews

Translated interview Guideline for teaching coordinators and administrative staff (Experts)

Family medicine clerkship

Version 05.07.2017

1. introduction

- Introduction of the interviewer; presentation of the purpose of the study. Reference to voluntariness, recording, and data protection and anonymized publication of data in professional journals.
- Explanation of the interview procedure
- Introduction of the interview partner: name, university, professional experience in current position

1. Initial question (Warm-up)

- In your opinion, what is the main goal of the family medicine clerkship?

1. Main questions

- How are teaching practices accredited at your university? Is it difficult to find enough teaching practices?
- What kind of feedback about teaching practices does your institution receive from students?
- Do any "problem practices" crystallize in the process? How would you define these? How do you deal with them? Are there consequences in case of negative events (training, warnings)?
- What structure is in place to avoid "problem practices"?
- In what situations have students sought a change in teaching practice? How have you dealt with this as an institution?
- What kind of feedback about students does your institute receive from teaching physicians?
- How do teaching practices handle problematic student behavior?
- How does the institute deal with problematic student behavior? Are there consequences (e.g. training, disciplinary action)?

1. Wrap-up question

- Have we forgotten anything / Is there anything you would like to add?
