# Supplementary material for: Are we prepared for the future? A mixed-method study on quality management in decentralized family medicine teaching
Source: Med Educ Online. 2021 May 11;26(1):1923114. doi: 10.1080/10872981.2021.1923114 (PMC8118471; doi:10.1080/10872981.2021.1923114)
Supplement: Supplemental Material [file ZMEO_A_1923114_SM9130.zip › Supplementary files/Supplement 2 Survey for review.docx]

# Survey among family medicine departments in Germany

Information and declaration of consent to the study

Dear study participant,

In this study, problematic and functioning areas of quality assurance in family medicine teaching at the respective location will be surveyed.

Furthermore, the individual processes that take place at the respective institutes in the event of problems with external courses are to be depicted.

The survey takes about 20 minutes to complete.

The main objective of this study is to show already existing processes for

Quality assurance of teaching during the family medicine clerkship, as well as the current status and possible goals in various areas of quality assurance in family medicine teaching.

The first part of the study consists of questions about quality assurance, communication, and the didactic training of teachers at your institute.

The second part of the questionnaire contains three case studies for which you will be asked further questions.

Finally, you will be asked to answer some general questions about yourself and the family medical institute where you work.

Please answer the questions in a way that corresponds to the current procedure in your institution.

**Declaration of consent**

I declare that I agree to the collection and processing of data within the scope of the study for anonymous evaluation.

I am aware that the results of this study will be published in medical journals, but in an anonymous form so that a direct reference to my person cannot be established.

I agree that my data will be processed as described here.

**1** This section is about quality assurance at your institute

**2 Do you actively pursue quality assurance* in teaching?**
* By quality assurance we mean structured regular measures which aim to influence measurable results (e.g. student evaluation).


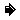
 Yes Please continue with question 3

| No | 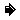Please continue with question 4 |
| --- | --- |

**3 Is the quality assurance based on a certain standard?**
(e.G. ISO 9000:2001)

No

| Yes, specifically: |  |
| --- | --- |

**4 What are you most satisfied with within your institution in terms of the organization of teaching?**
*by the institution we mean the family medicine department or institute with an affiliated chair at your university

**5 What area of your organization of teaching needs improvement?**

**6 What changes are you currently planning to meet these challenges?**

**7 What would help your institution most in implementing these improvements?**

**8 How many teaching practices are connected to your institution?**

less than 50 practices

| 51-100 |
| --- |

101-200

| 201-300 |
| --- |

more than 300 practices

**9 How many students are supervised by your institution in the family medicine clerkship per semester?**

less than 50 students

| 51-100 |
| --- |

101-200

| 201-300 |
| --- |

More than 300 students

**10 When does the family medicine clerkship take place for all students?**


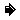
At a specific time during the semester, which is e.g. determined by
Please continue with question 11 the deanery

| At a time determined by the students | 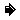Please continue with q.12. |
| --- | --- |

Other, specifically:

**11 When is this time?**

Only during the semester break

| Only during the semester |
| --- |

Both during the semester and the semester break

| Other, specifically |  |
| --- | --- |

**12 Do you use a standard for the selection of teaching practices (minimum requirements)?**

Yes, specifically:

| No |
| --- |

**13 Have you defined a didactic standard* for the family medicine clerkship?**
* for example a "standard curriculum" or "milestones"


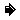
Yes Please continue with question 14.

| No |  |  | 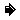 Please continue with question 18. |
| --- | --- | --- | --- |

**14 Is this didactic standard publicly accessible to interested practitioners?**

yes

| no |
| --- |

**15 If so, what do you think are the three most important learning opportunities* in this didactic standard?**
* by this we mean: the opportunity/method which has the greatest share in the learning success of the student in BP, which helps the students most in achieving the learning goals

**16 In your experience, what is the best way for teaching physicians to implement this didactic standard?**

**17 How do you check that the didactic standard is adhered to by the teaching practice?**

**18 Is there a performance assessment for the students in the family medicine clerkship??**


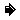
Yes Please continue with question 19

| No | 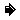Please continue with q. 20. |
| --- | --- |

**19 Where does this performance assessment take place?**

central (at your institution)

| decentralised (in practice) |
| --- |

both centralized and decentralized (e.g. workplace-based assessment and OSCE)

other, specifically:

**20** In this section you will be asked questions about communication with students.

**21 Do you systematically collect feedback from students about negative events in the family medicine clerkship*?**
* e.g. non-compliance with teaching standards, errors in content, unfriendly treatment by the teacher

Yes, specifically in the following manner:

| No |
| --- |

**22 Does it happen that students contact you because of negative events in the family medicine clerkship?**


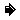
 Yes Please continue with question 23

| No | 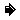please continue with question 25. |
| --- | --- |

**23 How many times do students contact you during the semester due to negative events?**

**24 How do students get in contact with you?**

**25** In this section you will be asked questions about communication with the teachers.

**26 Do you systematically collect feedback from teachers about negative events with students* in the family medicine clerkship?**
* e.g. unfriendliness, disinterest, lack of hygiene, serious gaps in knowledge

Yes, specifically in the following manner:

| No |
| --- |

**27 Does it happen that teachers contact you because of negative events in the family medicine clerkship?**


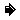
 Yes Please continue with question 28

| No | 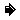Please continue with q. 31. |
| --- | --- |

**28 How many times do teachers contact you during the semester due to negative events with students?**

**29 How do teachers get in contact with you?**

**30 Do you want to tell us anything else about communication with teachers or students?**

**31** In this section you will be asked questions about the didactic training of the teachers at your institute.

**32 Do you regularly carry out didactic training measures for teachers?**


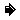
 Yes Please continue with question 33

| No | 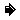Please continue with q. 39 |
| --- | --- |

**33 How often per year do these take place?**

1x per year

| 2x per year |
| --- |

3x per year

| 4x per year |
| --- |

More often than 4x per year

**34 How long do these events normally last each?**

1-2 hours

| 3-4 hours |
| --- |

More than 4 hours

**35 Is participation in these events obligatory?**


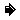
Yes Please continue with question 36.

| No | 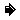Please continue with q.37. |
| --- | --- |

**36 What is the consequence of non-participation?**

**37 What incentives are available for this event?**

| Catering |  |
| --- | --- |

CME points

job fair for residents and/or final year students

| Monetary compensation |
| --- |

Other, specifically:

**38 In your experience, which training measure brings the greatest success* in the further training of teachers?**
* by this we mean if, after this training measure, teaching physicians significantly adapt their behavior to the standard or if student evaluation shows improvement.

**39 Case examples**

In the following part of the survey, three case studies are presented. We ask you to answer these case studies in a way that corresponds to the procedure in your institution.

**40** First case

A student calls you after the family medicine clerkship and reports that her GP teacher made derogative, unfair, and unmotivated comments about her in front of the nursing team. There had been sexualized comments.

You are unaware of past negative feedback on the teacher. To your best knowledge, the teacher is engaged, constructive, and popular among colleagues and students.

**41 How do you proceed in this situation?**

**42** Second case

On the 3rd day of the family medicine clerkship, a student writes an angry email complaining that he has no opportunity to work independently whatsoever at his current teaching practice.

The owner of the practice and teaching doctor has been around for a long time. His teaching performance is rather mediocre. Several student feedbacks indicate that independent work is hardly possible in this practice. The student wishes to change practices.

**43 How do you proceed in this situation?**

**44** Third case

A dedicated teacher, who has not been with your department for very long, calls you during the clerkship. She reports that her current student is acting irrationally. He has already used up his allowed absence time. Furthermore, he keeps leaving the room during the patient consultations.

He seems absent-minded in his contact with the nursing staff and during patient consultations. He avoids eye contact and has already been late several times. She is seriously concerned about the student's health. She is unsure if the student can even be certified as having successfully participated.

**45 How do you proceed in this situation?**

**46** Now we are almost at the end of the questionnaire. We now ask you for some data about your institute and your position

**47 How long have you been working at your institution in your current position?**

0-3 years

| 4-6 years |
| --- |

7-10 years

| more than 11 years |
| --- |

**48 What is your position at your family medicine department?**

administrative position (e.g. teaching secretariat)

| management position (e.g. chair holder, teaching coordinator) |
| --- |

**49 How many employees work at your family medicine department?**
Please indicate the number of employees irrespective of full-time or part-time employment

1-5 employees

| 6-10 employees |
| --- |

11-15 employees

| 16-20 employees |
| --- |

More than 20 employees

**50 How many doctors in private practice work for you as lecturers*?***by lecturers we mean doctors who actively participate as lecturers in courses. Teaching practices are only included if, in addition to a contract as a teaching practice, they also have a contract as an assistant lecturer

0-10 doctors

| 11-20 doctors |
| --- |

21-30 doctors

| 31-40 doctors |
| --- |

41-50 doctors

| more than 50 doctors |
| --- |

**51 For how many years has your family medicine department been with an independent chair?**

0-10 Years

| 11-20 Years |
| --- |

21-30 Years

| 31-40 Years |
| --- |

41-50 Years

| No chair established (yet) |
| --- |

**52 Would you like to tell us anything else about quality assurance in teaching?**

**53** Thank you for participating!
