# Supplementary material for: Are we prepared for the future? A mixed-method study on quality management in decentralized family medicine teaching
Source: Med Educ Online. 2021 May 11;26(1):1923114. doi: 10.1080/10872981.2021.1923114 (PMC8118471; doi:10.1080/10872981.2021.1923114)
Supplement: Supplemental Material [file ZMEO_A_1923114_SM9130.zip › Supplementary files/Supplement 3 Coding Frame.docx]

| Main category | Category | Subcategory 1st level | Subcategory 2nd level |
| --- | --- | --- | --- |
| Teacher - student interaction in the clerkship |  |  |  |
|  | **Problems in the clerkship, in the 1:1 supervision** |  |  |
|  |  | **Problematic student behavior** | **Failure to meet minimum attendance time** |
|  |  |  | **tardiness** |
|  |  |  | **inappropriate physical appearance** |
|  |  |  | **disinterest** |
|  |  |  | **lack of commitment** |
|  |  |  | **Inappropriate student interaction with patients or practice team** |
|  |  |  | **Behavior or characteristic of student that is challenging in terms of teaching or dealing with patients.** |
|  |  |  |  |
|  |  | **Problematic behavior by teaching physicians** | **Definition of poor treatment** |
|  |  |  | **case reports on student mistreatment** |
|  |  |  | **Definition of "Problem practices"** |
|  | Expectations |  |  |
|  |  | Expectations of practices |  |
|  |  | Desireable student prerequisites and attributes |  |
| Structure and organisation of the clerkship |  |  |  |
|  | **Teaching environment** (General conditions, practice conditions) |  |  |
|  |  | Conditions at practice level | Access route |
|  |  |  | facilities |
|  |  |  | Structure and organisation |
|  |  | Workload of the teaching physicians |  |
|  |  | Clerkship duration |  |
|  | Tasks of the institute of familiy medicine |  |  |
|  |  | student supervision by the institute |  |
|  |  | Responsibilities |  |
|  |  | Provision of teaching materials, accompanying seminars |  |
|  |  | Distribution of the students to the practices, logistics, travel expenses |  |
|  |  | Recruitment of new teaching practices |  |
|  |  | **Accreditation procedures and contracts with teaching practices** |  |
|  |  | **Re-accreditation** |  |
|  |  | **Support of the GP teachers by the institute** | **Continuing education, coaching, training of GP teachers** |
|  |  |  | GP teacher appreciation by the institute |
|  | Information teaching practice, communication institute - teaching practice |  |  |
|  |  | expectations on GP teachers, |  |
|  |  | teaching mission, |  |
|  |  | clerkship curriculum |  |
|  |  | practice team training |  |
|  |  | communicating what can be expected of a medical student |  |
|  |  | communicating what a medical student is allowed to do |  |
|  | Student information |  |  |
|  | Curriculum |  |  |
|  |  | coexistence of clinical elective and clerkship |  |
|  |  | role of family medicine in the medical curriculum |  |
|  |  | compulsory nature of the clerkship |  |
|  |  | masterplan 2020 |  |
|  | Problem solving strategies by the institute |  |  |
|  |  | **dealing with problem practices** |  |
|  |  | change practices |  |
|  |  | **dealing with problematic student behavior** |  |

Supplementary Table 1: Summarized coding frame based on expert interviews. Categories of particular interest for the survey were highlighted in bold text.
